# Single cell transcriptomics analyses reveal functional heterogeneity and anti-tumor role of mast cells in esophageal squamous cell carcinoma

**DOI:** 10.3389/fimmu.2026.1761865

**Published:** 2026-07-30

**Authors:** Yiren Huang, Zheyi Chen, Bingqian Zhou, Shiyu Chen, Yongyu Chen, Qiu Pan, Weinan Zhu, Chenyu Ma, Shihong Chen, Lisong Shen, Ju Qiu, Yingxia Zheng

**Affiliations:** 1 Department of Laboratory Medicine, Xin Hua Hospital, Shanghai Jiao Tong University School of Medicine, Shanghai, China; 2 College of Health Science and Technology, Shanghai Jiao Tong University School of Medicine, Shanghai, China; 3 Institute of Artificial Intelligence Medicine, Shanghai Academy of Experimental Medicine, Shanghai, China; 4 CAS Key Laboratory of Tissue Microenvironment and Tumor, Shanghai Institute of Nutrition and Health, University of Chinese Academy of Sciences, Chinese Academy of Sciences, Shanghai, China

**Keywords:** esophageal squamous cell carcinoma, mast cells, single-cell RNA sequencing, spatial transcriptomics, TNF

## Abstract

**Introduction:**

Mast cells (MCs) play important roles in allergic reactions and tissue homeostasis; however, their functions in esophageal squamous cell carcinoma (ESCC) remain controversial. Understanding the heterogeneity and functional states of MCs in ESCC is essential for elucidating their roles in tumor progression and immune regulation.

**Methods:**

single-cell RNA sequencing (scRNA-seq) data from 84 ESCC samples across four independent cohorts were analyzed to identify MC subtypes, which were further validated using bulk RNA-seq and immunofluorescence staining. Functional assays were performed to assess the effects of stem cell factor (SCF)-stimulated MCs on ESCC cell proliferation, migration, and apoptosis. Spatial transcriptomics were used to investigate MC interactions within the tumor microenvironment (TME).

**Results:**

MC abundance was significantly reduced in ESCC tissues compared with normal esophageal tissues, as confirmed by bulk RNA-seq and IHC analyses. scRNA-seq revealed five distinct MC subtypes in ESCC: T-type-activated MC, T-type-resting MC, TC-type-activated MC, TC-type-resting MC and proliferating MC. Resting MCs localized to normal and stromal regions, whereas activated MCs were enriched in stromal and tumor regions, with their proportion significantly elevated in tumor tissues. SCF activated MCs via the c-Kit pathway, promoting TNF-α release and tumor cell apoptosis. Spatial and cell-cell communication analyses revealed extensive MC interactions with stromal and immune cells. Activated MC abundance positively correlated with T-cell infiltration and favorable ESCC prognosis.

**Conclusions:**

Our findings demonstrate that MCs exhibit distinct functional states in ESCC and regulate tumor progression. T-type-activated MCs, characterized by enhanced TNF-α expression, may contribute to anti-tumor immunity. These findings provide new insights into MC heterogeneity in ESCC and support their potential relevance in immunotherapy.

## Introduction

Esophageal cancer (EC) is one of the most prevalent malignancies of the gastrointestinal tract, characterized by high lethality, marked invasiveness, and ranks as the sixth leading cause of cancer-related deaths globally (1). Based on histopathological features, EC is classified into esophageal squamous cell carcinoma (ESCC) and esophageal adenocarcinoma (EAC), with ESCC accounting for approximately 90% of cases in East Asia (2, 3). Due to its insidious onset and nonspecific clinical manifestations, most ESCC patients are diagnosed at an advanced stage, leading to a poor 5-year overall survival rate (4). Nowadays, neoadjuvant therapy, particularly immune checkpoint blockade (ICB) has emerged as a key treatment strategy for patients with advanced ESCC (5). However, the therapeutic efficacy of ICB in ESCC remains limited, for advanced or recurrent ESCC, PD-1/PD-L1 inhibitor monotherapy only elicits a response (complete or partial remission) in approximately 14.4% to 20% of patients (6, 7). Therefore, a comprehensive understanding of the complex interactions among diverse cell populations and molecular components within the tumor microenvironment (TME), and the identification of novel immunotherapeutic targets is required in ESCC treatment.

The growth of single-cell RNA sequencing (scRNA-seq) allows for the detailed characterization of gene expression profiles at the single-cell level, which in turn provides insights into cellular heterogeneity, rare cell types, and the diversity of cell subpopulations (8). In recent years, the phenotypic and functional landscapes of lymphocytes and myeloid cells in the context of ESCC have been progressively delineated (9, 10), the dominant cell populations include exhausted T cells, exhausted NK cells, regulatory T cells, alternatively activated macrophages, and tolerogenic dendritic cells, all of which contribute to the immunosuppressive landscape of the tumor microenvironment (TME). However, research on MCs in the context of ESCC is limited and has not yet been unified (11, 12).

Mast cells (MCs) are innate immune cells that are extensively found in human tissues and organs. They are best known for playing crucial roles in allergic diseases like atopic dermatitis, asthma, and allergic rhinitis (13). Upon stimulation by allergens, cytokines, or other microenvironmental signals, MCs can undergo activation and release a broad range of bioactive mediators, including histamine, cytokines, chemokines, and proteases, thereby regulating immune responses and tissue homeostasis (14). Accumulating evidence has highlighted the critical roles of MCs within the tumor microenvironment, where they participate in angiogenesis, extracellular matrix remodeling, immune regulation, and tumor progression (15, 16). However, the role of MCs in cancer remains highly context-dependent and controversial, as they may exert either tumor-promoting or tumor-suppressive functions depending on tumor type, disease stage, and local immune contexture (17, 18). For example, in prostate and lung cancers, MCs promote tumor progression during early disease stages but exhibit certain inhibitory effects in advanced disease (19, 20). In colorectal cancer, MCs promote small intestine cancer in a tumor stage-specific and arginine-dependent manner, yet they can also derive LTB4 to recruit CD8^+^ T cells for anti-tumor activity (21). These findings collectively suggest substantial functional heterogeneity of MCs across different tumor contexts. In ESCC, the functional significance of MCs remains incompletely understood. Previous studies have reported that increased mast cell density at the invasive margin of ESCC is associated with enhanced tumor aggressiveness and poor patient survival (22). In contrast, another study demonstrated that MCs in ESCC highly express IL-17, thereby promoting the activation of CD8^+^ T cells and macrophages and exerting anti-tumor effects (12). The contradictory findings imply that MCs in ESCC may exhibit distinct functional states or subtype-specific activities within the TME. Specifically, the subpopulation classification of MCs, their potential function, the crosstalk between MCs and other cellular components in the TME, as well as the correlation between MCs and tumor prognosis in ESCC, have not been clearly elucidated. Thus, more study is required to develop a more comprehensive understanding of the functional roles of those MCs in ESCC.

Our study focuses on the functional heterogeneity and anti-tumor mechanisms of mast cells within the tumor microenvironment of ESCC. In this work, we integrate single-cell RNA sequencing and spatial transcriptomics with comprehensive functional assays to systematically characterize MC heterogeneity and decipher the anti-tumor functions of MCs in ESCC. We identify predominant MC cellular states across ESCC lesions, dissect their immunological characteristics and tumor-related properties, and elucidate their crosstalk with other cellular components in the tumor microenvironment. Notably, our findings distinguish resting and activated MC subpopulations and uncover their pivotal implications in immune modulation and ESCC malignant progression.

## Methods

### Materials

The study used a number of publicly available datasets, including five bulk RNA-sequencing (bulk RNA-seq) datasets and five scRNA-seq datasets (GSE160269 (9), GSE145370 (10), GSE188900 (23), GSE196756 (24), and GSE221561 (25)). The bulk RNA-seq datasets comprised data from TCGA-ESCA and four gene expression comprehensive (GEO) microarray datasets (GSE20347, GSE23400, GSE44021, GSE53625). Transcriptomic and clinical information from GEO datasets was obtained from the GEO database (https://www.ncbi.nlm.nih.gov/geo/). Detailed patient demographic information, tissue origin, sample type, and dataset characteristics were summarized in **Supplementary Table 1**.

### Sample collection and processing

The analyzed samples primarily consisted of freshly resected primary ESCC tumor tissues and paired adjacent normal esophageal tissues collected during surgery. Adjacent normal tissues were generally obtained at least 5 cm away from the tumor margin. Most patients included in these cohorts had not received chemotherapy, radiotherapy, or immunotherapy prior to surgical resection, except for a subset of samples from GSE221561, which included patients receiving neoadjuvant therapy. Tumor and normal tissue identities were confirmed by histopathological examination in the original studies. Fresh tissue samples were processed into single-cell suspensions according to the protocols described in the corresponding publications and sequenced mainly using the 10 × Genomics platform. All datasets included in this study were generated with informed consent and approval from the corresponding institutional ethics committees.

### Cell culture

The mouse ESCC cell line mEC25 was maintained in our laboratory (Shanghai, China). The mouse mast cell line P815 was obtained from Pricella (Wuhan, China). Both cell lines were cultured in DMEM medium (Hyclone) supplemented with 10% fetal bovine serum (FBS, Hyclone) and 1% antibiotic mixture (100 U/mL penicillin and 100 μg/mL streptomycin) to form complete medium. Both cell lines were cultured in a 37 °C, 5% CO_2_ incubator. Cell growth was monitored regularly, and the medium was changed every 2–3 days.

### Total RNA extraction and qRT-PCR

Total RNA was isolated and extracted from mEC25 and P815 cell lines using Trizol reagent (Invitrogen, 15596018CN). The RNA was then reverse transcribed into cDNA using 5 × HiScript III qRT SuperMix (Vazyme, R323). Gene expression levels were then quantified by qRT-PCR using Hieff^®^ qPCR SYBR Green Master Mix (YEASEN, 11203ES08). GAPDH served as the internal control. Relative mRNA expression was calculated using the 2^-ΔΔCt^ method. The primer sequences are provided as following. KIT forward: 5’-GGC, CTC, ACG, AGT, TCT, ATT, TAC, G-3’; reverse: 5’-GGG, GAG, AGA, TTT, CCC, ATC, ACA, C-3’; TNF forward: 5’- CAG, GCG, GTG, CCT, ATG, TCT, C-3’; reverse: 5’-CGA, TCA, CCC, CGA, AGT, TCA, GTA, G-3’; GAPDH forward: 5’-GGT, GAA, GGT, CGG, TGT, GAA, CG-3’; reverse: 5’-CTC, GCT, CCT, GGA, AGA, TGG, TG-3’.

### Cell co-culture, apoptosis and survival analyses

P815 cells and mEC25 cells were cultured in complete DMEM medium for 24 hours. The medium was then replaced with serum-free DMEM for an additional 24 hours, and the supernatants were collected. To investigate the role of KITLG-cKIT signaling in regulating MC-induced apoptosis, P815 cells were pretreated with mouse recombinant SCF (100 ng/mL, MedChemExpress, HY-P70555) or the specific inhibitor iSCK03 (10 μM, MedChemExpress, HY-101443) for 24 hours before co-culturing with mEC25 cells. Apoptotic cells were measured using the Apoptosis Detection Kit Annexin V-FITC/PI (YEASEN, 40302ES) according to the manufacturer’s instructions. Cell viability analysis was performed using the CCK-8 Cell Proliferation Assay Kit (Dojindo Laboratories, Japan).

### Transwell migration and invasion assays

In order to evaluate the migration and invasion abilities of the cells, we used a 24-well Transwell (Corning, 3422) with 8 μm pores. Co-cultured mEC25 cells (1×10^5^ cells) were seeded in 200 μL of FBS-free medium into the upper chamber. In the lower chamber, 700 μL of complete medium containing 10% FBS was added. After incubation at 37 °C for 48 hours, non-migrated cells were removed, and migrated cells were fixed and stained with crystal violet. Cell counting in five random fields were performed under a microscope. For Matrigel (Corning, 356231) invasion assays, follow the same procedure as for migration assays.

### Cell scratch assay

Scratch assays were performed to assess the migration potential of cancer cells. mEC25 cells were seeded into 6-well plates. Upon reaching 80% confluence, a pipette tip was placed perpendicular to the bottom of the plate and used to create a scratch in the cell monolayer. Results were photographed at 0 h, 24 h, and 48 h. Gap distances were quantified using ImageJ software.

### Immunohistochemistry and immunofluorescence staining

Paraffin-embedded human tissue sections were obtained from Xinhua Hospital, Shanghai Jiao Tong University School of Medicine, under an approved Institutional Review Board protocol. Tissue sections were incubated at 62 °C for 1 hour, deparaffinized in xylene, and sequentially rehydrated in 100%, 95%, and 70% ethanol. Endogenous peroxidase activity was blocked using hydrogen peroxide blocking solution for 10 minutes, followed by three washes with PBS. Antigen retrieval was performed by immersing sections in boiling Tris-EDTA buffer (1 mmol/L, pH 9.0) for 15 minutes. After PBS washing, sections were blocked with 5% bovine serum albumin (BSA) for 20 minutes. For immunohistochemistry (IHC), sections were incubated with primary antibodies overnight at 4 °C, followed by incubation with HRP-conjugated secondary antibodies at 37 °C for 30 minutes. Signals were visualized using DAB substrate, and nuclei were counterstained with hematoxylin. For immunofluorescence staining, sections were incubated with primary antibodies overnight at 4 °C, followed by incubation with fluorescent secondary antibodies at 37 °C for 30 minutes. TSA amplification kits (RunnerBio) were used for signal amplification where indicated. Nuclei were counterstained with DAPI. Images were acquired using a digital slide scanner (PANNORAMIC SCAN II, 3DHISTECH) and analyzed using SlideViewer and ImageJ software.

### scRNA-seq data processing

This study analyzed one scRNA-seq dataset (GSE221561) and performed an integrated analysis of four publicly available ESCC scRNA-seq datasets (GSE145370, GSE160269, GSE188900, and GSE196756). Quality control was performed using the “Seurat” package. Genes detected in fewer than 3 cells were excluded. Cells with fewer than 200 detected genes, more than 7000 detected genes, mitochondrial gene proportions greater than 20%, or total UMI counts fewer than 1000 were considered low-quality cells and removed from downstream analyses. To reduce batch effects among datasets, the “Harmony” package was used for data integration. The “Seurat” package was used for cell normalization and regression to obtain a scaled barcode-gene matrix. The top 2000 differentially expressed genes were identified and used for principal component analysis (PCA). The top 50 PCA components were utilized in the FindNeighbors function. The FindClusters function identified cell clusters, visualized via UMAP plots. The FindAllMarkers function calculated cluster marker genes to characterize mast cells and other cell types. To further identify mast cell subpopulations, unsupervised clustering was performed on mast cell subpopulations. Potential doublets were identified and removed using the “DoubletFinder” package during both the initial integrated dataset preprocessing and mast cell subclustering analyses. Clusters exhibiting mixed lineage marker expression were additionally excluded from downstream analyses.

### Defining MC subtype

In order to characterize the differences among various MC subsets, we used the “AddModuleScore” function implemented in the Seurat package. These scores were calculated based on the average expression of genes associated with a particular subtype or functional state. Initially, MCs were stratified into T-type MC (MC_T) and TC-type MC (MC_TC) according to the expression patterns of canonical subtype markers (TPSAB1, CPA3, CMA1 and CTSG) (26). Subsequently, activation scores were calculated separately within each subtype using their corresponding activation-associated gene signatures. Based on these scores, MC_T cells were further classified into T-type activated MCs and T-type resting MCs, whereas MC_TC cells were classified into TC-type activated MCs and TC-type resting MCs. The previously reported MC activation signature (27) was used as the basis for defining subtype-specific activation gene sets (**Supplementary Table 6**).

### Functional enrichment analysis

GO enrichment analysis was performed between tumor and normal MCs via Gene Ontology Biological Process (GO BP) gene sets.

### Tissue distribution analysis

For the characterization of meta-clusters’ tissue distribution, odds ratios (OR) were calculated using normal tissue as the reference group to quantify the tissue localization preference of each cluster (28). Specifically, an OR > 1.5 indicates that the given cell population has a strong preference for enrichment in tumor tissue, while an OR < 0.5 suggests a marked preference for normal tissue. Values between 0.5 and 1.5 represent no obvious tissue distribution bias.

### Cell communication analysis

To investigate interactions between MCs and other major cell types, we employed CellChat and CellphoneDB methodologies to identify inferred ligands and receptors based on their expression in each cell. The CellChat (version 1.6.1) was primarily used to characterize the global cell–cell communication landscape, including the number and strength of inferred ligand–receptor interactions among different cell types. CellPhoneDB (version 2.0.6) was subsequently used to identify and analyze specific ligand–receptor signaling pathways based on clustering annotations.

### TCGA database and survival analysis

Survival analysis were performed using the TIMER3 (https://compbio.cn/timer3/) and the GEPIA Webserver (http://gepia2.cancer-pku.cn/). *P* values were calculated using Kaplan-Meier methods with a log-rank test.

### Spatial transcriptomic analysis

Fresh frozen ESCC tissues were embedded in optimal cutting temperature (OCT) compound and sectioned according to the manufacturer’s protocol for 10 × Genomics Visium spatial transcriptomics. Tissue sections were selected based on hematoxylin and eosin (H&E) staining to ensure high-quality morphology. Following tissue optimization, spatial gene expression library preparation and sequencing were performed according to the manufacturer’s instructions. Raw spatial transcriptomic data were processed and normalized using the “SCTransform” function in the “Seurat” R package. Simplification and clustering were performed using “RunPCA” and “RunUMAP”. After merging similar clusters, we identified normal, stromal, and tumor regions within ESCC samples. To annotate cell types in spatial spots, we applied the “SPOTlight” deconvolution algorithm based on single-cell RNA-seq reference data. In addition, to integrate mast cell subpopulation signatures with spatial transcriptomic data, we derived the top ten differentially expressed genes from total activated and resting mast cell clusters identified in the single-cell dataset. Gene module scores for activated and resting mast cell signatures were calculated using the “AddModuleScore” function in Seurat and projected onto spatial maps to evaluate their spatial distribution.

### Deconvolution analysis

SPOTlight analysis was performed for deconvolution analysis as Elosua‐Bayes et al. reported (29).

### Statistical analysis

The statistical analysis in this study was conducted using R (version 4.2.2), GraphPad Prism (version 9), and Python (version 3.8). The student’s unpaired two-tailed t-test was used for comparisons between two groups; analysis of variance test was used for comparisons of more than two groups. Statistical significance was denoted with (*P < 0.05; **P < 0.01; ***P < 0.001) in the Figure. Survival analyses was estimated by the Kaplan-Meier method and compared by log-rank tests.

## Results

### Single-cell atlas of ESCC

**Figure 1:**
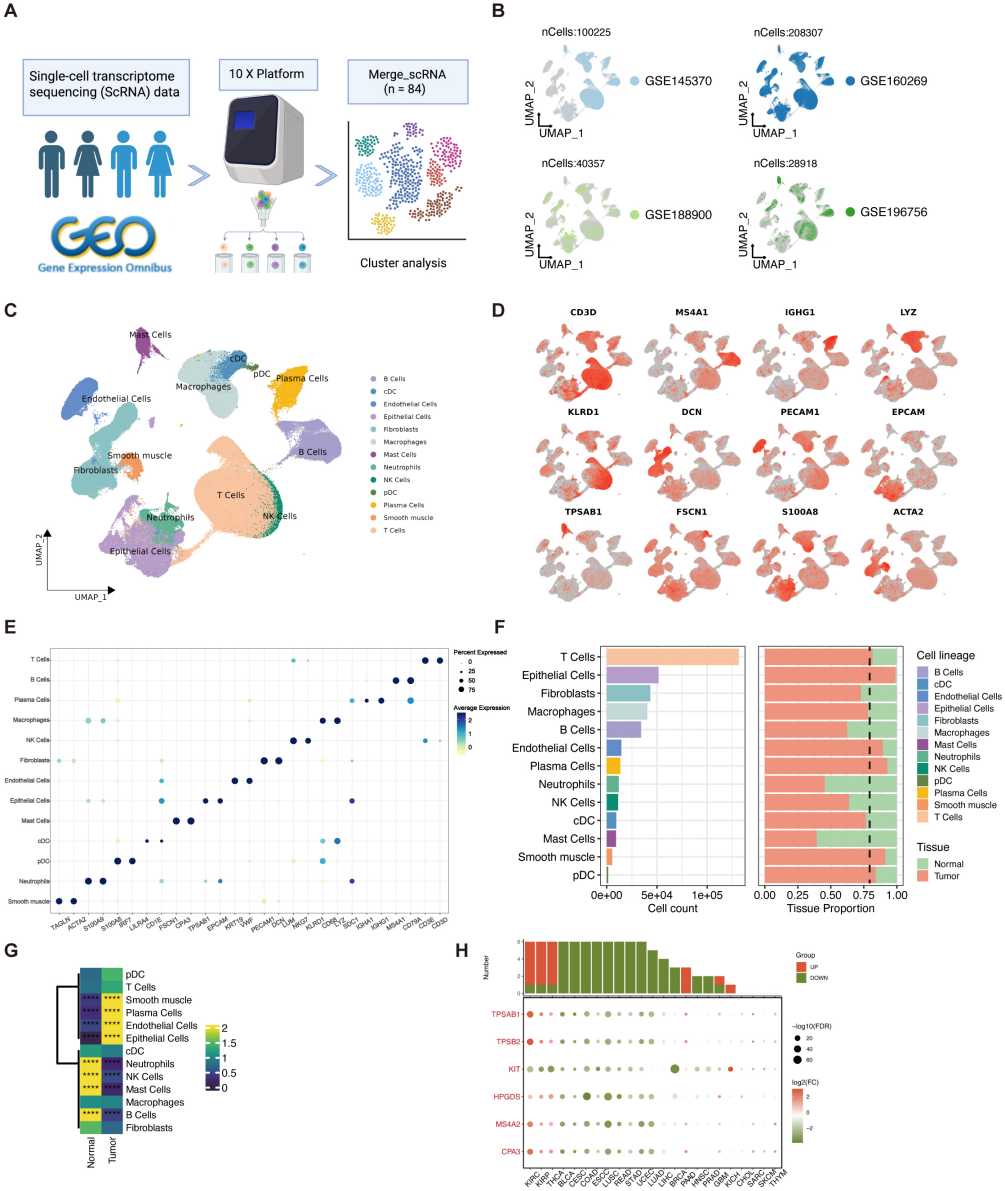
single-cell atlas of ESCC. **(A)** scRNA-seq data from GEO were integrated into aMerge_scRNA object. **(B)** UMAP distribution of single cells from the four datasets withinMerge_scRNA. **(C)** UMAP clustering of thirteen major cell types. **(D)** UMAP plots showed marker gene expression for major cell types. **(E)** Dot plot displayed marker genes of each major cell types in our study. **(F)** Histogram showed the cell counts and tissue proportion of major cell types. **(G)** The odds ratio (OR) analysis of major cell types between Normal and Tumor. Fisher’s exact test was applied on this contingency table. **(H)** Dot plot showed the expression of MC signature genes in each cancer (Normal vs. Tumor). Histogram showed the number of genes with statistical significance (upper). Red represented an increase in tumor expression, green represented a decrease in tumor expression, and only *P* values < 0.05 are displayed. ****P < 0.0001.

To investigate the role of mast cells in ESCC progression, we first integrated four ESCC single-cell datasets of untreated ESCC samples (GSE145370, GSE160269, GSE188900, and GSE196756) (**Supplementary Table 1**); forming a multi-center cohort comprising 84 ESCC samples (**Figures 1A, B**). Each ESCC single-cell dataset underwent separate single-cell processing workflows beforehand. Harmony algorithm was employed to effectively mitigate batch effects across datasets and samples, thereby optimizing sample distribution and minimizing the impact of experimental variability on results. Each cluster contained cells from all four datasets, indicating no significant dataset-specific batch effects (**Supplementary Figure 1A**). Following dataset integration, rigorous quality control was performed, and low-quality cells failing to meet standards were removed, finally, a total of 377,807 cells (including tumor and adjacent normal tissue) were selected for further analysis (**Figure 1C**; **Supplementary Figure 1B**). Cells were annotated using their characteristic markers, ultimately identifying main cell types including T cells (CD3D, CD3E), B cells (CD79A, MS4A1), plasma cells (SDC1, IGHG1), macrophages (CD68, LYZ), NK cells (NKG7), mast cells (TPSAB1, TPSB2), DC cells (IRF7, CD1E), neutrophils (S100A8, S100A9), fibroblasts (DCN, LUM), endothelial cells (PECAM1, VWF), and epithelial cells (EPCAM, KRT19) (**Figures 1D, E**; **Supplementary Table 2**).

Comparing the distribution of each cell type between tumor and normal tissues, we observed that the integrated dataset contained a higher proportion of tumor-derived cells overall (78.8% versus 21.2% normal-derived cells). Relative to this expected proportion, we found that T cells, plasma cells were more abundant in tumors across the integrated dataset, whereas mast cells, B cells, NK cells, and neutrophils were more abundant in normal tissues (**Figure 1F**), which was consistent with previous single-center cohort studies (10). Besides, we found that the majority of cells were CD45^+^ immune cells, whereas CD45^-^ epithelial cells, fibroblasts, and endothelial cells accounted for only 13.64%, 11.47%, and 3.83% of the total cell population. Interestingly, mast cells account for approximately 3.52% of the total CD45^+^ immune cells in the ESCC tumor microenvironment (**Figure 1F**; **Supplementary Figure 1C**, S1D). To further analyze the tissue preferences of different immune cell types, we performed odds ratio (OR) analysis (28). Accordingly, OR values < 0.5 indicate enrichment in normal tissues, whereas OR values > 1.5 indicate enrichment in tumors. Results revealed that mast cells, B cells, NK cells, and neutrophils exhibited strong distribution preferences in normal tissue, while plasma cells appeared to be enriched in tumors (**Figure 1G**). To identify MC signature genes in ESCC for subsequent analyses, we obtained the intersection of MC marker genes from three single-cell RNA sequencing datasets (GSE145370, GSE160269, and GSE196756). We established a stringent gene selection criterion (*P* value < 0.01, log2-fold change (log2FC) > 1, PCT1 > 0.7, and PCT2 < 0.3). Considering that LTC4S is commonly expressed in basophils, we finally selected TPSAB1, TPSB2, KIT, HPGDS, CPA3, and MS4A2 as the definitive marker genes for mast cells (**Supplementary Figure 1E**; **Supplementary Table 3**). Additionally, we use six MC marker genes to assess MC density across a large number of RNA-seq samples from tumors and normal tissues in the TCGA database. We found that MC signature genes were elevated only in KIRC, KIRP, and THCA, while they were downregulated in BLCA, CESC, COAD, ESCC, LUSC, READ, STAD and UCEC. (**Figure 1H**) (Abbreviations of the cancer are shown in **Supplementary Table 4**). Collectively, our data showed that the density of MCs is reduced across the majority of tumors.

### Characteristics of MCs and reduced MC density in ESCC

**Figure 2:**
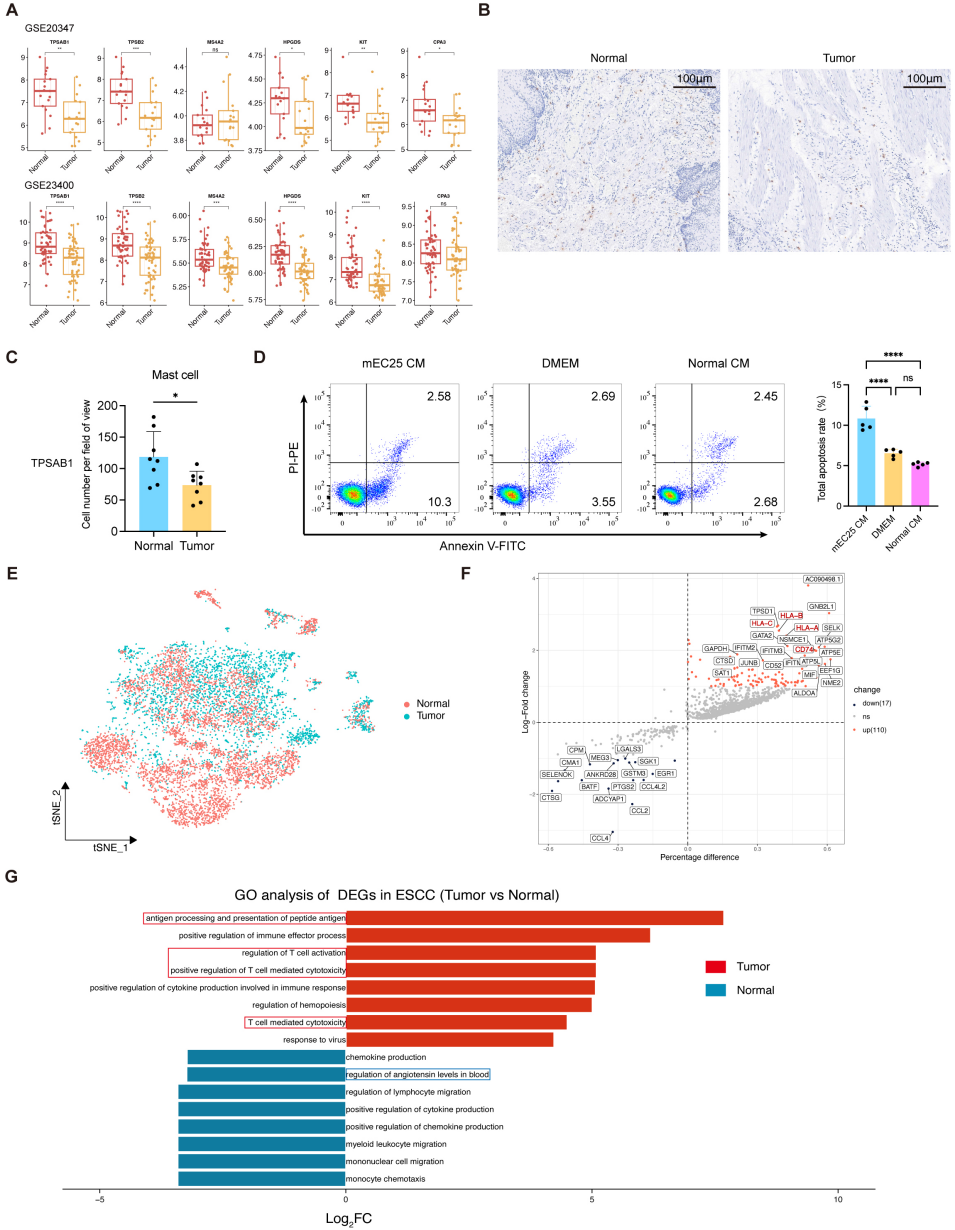
Characteristics of MC and reduced MC density in ESCC. **(A)** Box plots showed the expression of MC marker genes in 2 bulk RNA-seq cohorts of ESCC patients (Normal vs Tumor). **(B)** IHC stained of TPSAB1(Mast cell) in human ESCC tissue and paired NC tissue. **(C)** Bar chart displayed the average cell number in each field(*n* = 8). *P* values was calculated by two-tailed Student’s *t*-test. **(D)** mEC25 CM was collected and then co-cultured with P815, the apoptosis of P815 after co-cultivation was detected by flow cytometry(*n* = 5). Data was showed as the mean ± SD. *P* values was calculated using one-way analysis of variance. **(E)** TSNE plot of Mast cells scRNA data stratified by tissue types. **(F)** Volcano plot showing the DEGs between ESCC MCs and NC MCs from our study. Each red dot denoted an individual gene with an adjusted *P* values < 0.01 and fold change > =1, two-sided Wilcoxon test. All the genes were listed in **Supplementary Table 5**. **(G)** Enriched pathways in ESCC MCs and NC MCs according to the GO pathway enrichment analysis. *P < 0.05; **P < 0.01; ***P < 0.001; ****P < 0.0001; ns, not significant.

To further characterize alterations in mast cell density in ESCC, we analyzed several bulk RNA-seq cohorts and identified at least five mast cell marker genes that exhibit reduced expression in tumor tissues (**Figure 2A**; **Supplementary Figure 1F**). This finding was further corroborated by our IHC results from paired ESCC and normal samples, which demonstrated a significant reduction in mast cell density within ESCC (**Figures 2B, C**). Meanwhile, immunofluorescence analysis showed markedly higher fluorescence intensity of mast cells in normal tissues (**Supplementary Figure 2A**). We hypothesized that the reduction in MCs may be due to the ESCC TME, potentially due to factors originating from tumor cells. To test this hypothesis, we treated P815 mast cell line with cell culture supernatant derived from mouse ESCC cell line mEC25, and observed increased P815 cell apoptosis (**Figure 2D**; **Supplementary Figure 2B**). This finding indicated that esophageal cancer cells in the TME may exert a negative effect on mast cell survival, which in turn contributes to the subsequent reduction in mast cell density.

To better understand the characteristics of MCs in ESCC TME, we performed dimensionality reduction and clustering analysis on MC populations extracted from ESCC patients. First, we examined the cell distribution maps stratified by tumor and adjacent normal tissues (**Figure 2E**) to visualize inter-tissue MC distribution patterns; this was followed by differential gene expression (DEGs) analysis between the two tissue-derived MC populations (**Supplementary Table 5**). We discovered the number of DEGs was significantly higher in tumor tissues than in normal tissues. (*P* value < 0.05, log2FC > 0.25). Volcano plots revealed that genes involved in the antigen presentation related genes, including HLA-A, HLA-B, HLA-C, and CD74 were significantly increased in tumor tissue MCs (**Figure 2F**), suggesting that MCs within tumors may exert anti-tumor effects. GO analysis revealed that pathways associated with antigen presentation and T cell activation were significantly enriched in MCs from ESCC tumor tissue, while pathways related to angiogenesis and chemokines were enriched in MCs from normal tissue (**Figure 2G**). Taken together, these findings highlighted the functional complexity of MCs in the context of ESCC progression.

### Heterogeneity of MCs in ESCC

**Figure 3:**
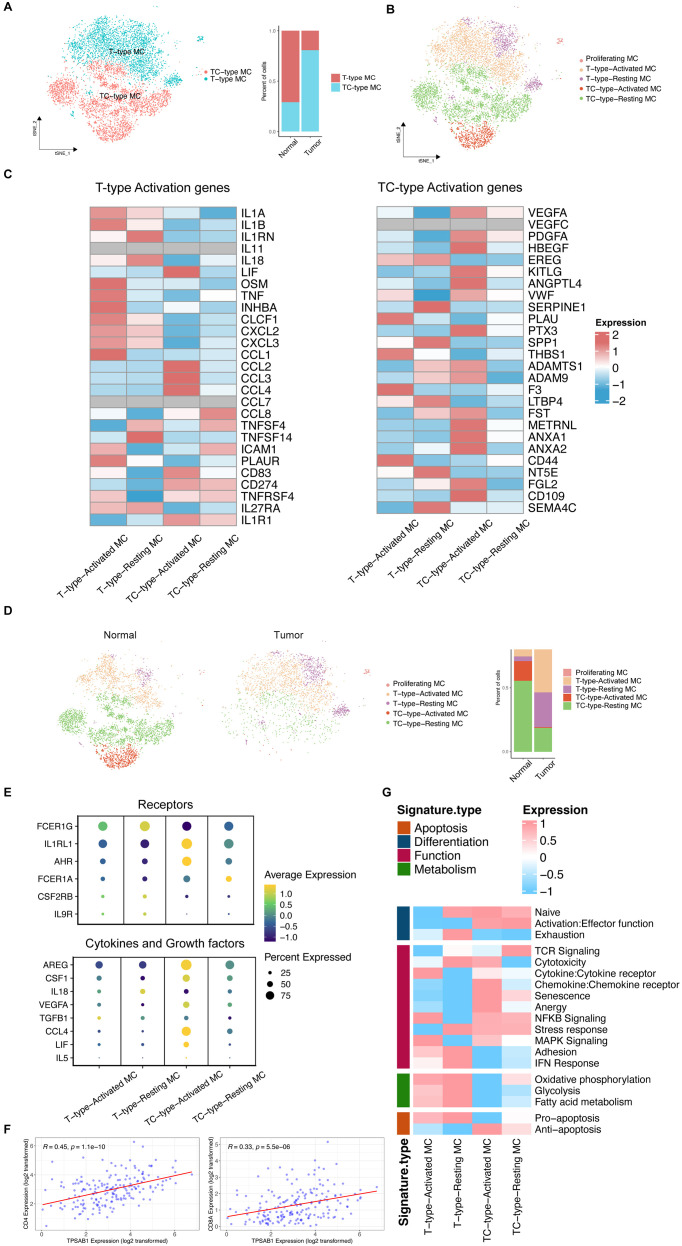
Heterogeneity of MCs in ESCC. **(A)** Left: TSNE plots showed MCs color-coded by 2 clusters, namely “T-type MC” and “TC-type MC”. Right: Bar charts showed the proportion of MC_T and MC_TC in different tissues. **(B)** TSNE plots showed MCs color-coded by 5 subclusters, namely “T-type-activated MC”, “T-type-resting MC”, “TC-type-activated MC”, “TC-type-resting MC”, and “Proliferating MC”. **(C)** Heatmap showing the expression patterns of T-type and TC-type activation genes in the four MC subpopulations. **(D)** Left: TSNE plots showed distinct separation of MC subtypes between normal and tumor tissues. Right: Bar charts showed the proportion of MC subtypes in different tissues. **(E)** Dotplots showing the Receptors, Cytokines and Growth factors-related gene expression in MC clusters. **(F)** Correlation analysis of TPSAB1, CD4, and CD8A expression in TCGA-ESCC dataset (Spearman test). **(G)** Heatmap showing the correlation between MC subsets and CD8^+^ T cell function.

To further explore the heterogeneity within MCs, we performed subpopulation analysis of 9,266 MCs identified from the integrated ESCC dataset. The subclusters enriched for both MC marker genes, myeloid marker genes and T cell-associated genes were designated as “Non-MCs” (**Supplementary Figure 2C**). Considering the potential impact of the mixed cell stage, these cells were excluded from subsequent analyses. Previous studies have demonstrated that human MCs can be broadly classified into two major subtypes, MC_T and MC_TC, according to their protease expression profiles (30, 31). Accordingly, MCs were first stratified into “T-type MCs” (MC_T), characterized by high TPSAB1 and CPA3 expression with low CMA1 and CTSG expression, and “TC-type MCs” (MC_TC), characterized by co-expression of TPSAB1, CPA3, CMA1, and CTSG (**Figure 3A**; **Supplementary Figures 2D, E**). T-type MCs were considered analogous to mucosal MCs and were generally associated with inflammatory and immunoregulatory responses, whereas TC-type MCs resembled connective tissue MCs and were more closely linked to tissue remodeling and angiogenesis. Next, we categorized the MCs based on the expression of previously published lists of genes indicating cell activation (27) (**Supplementary Figure 2F**; **Supplementary Table 6**). Based on these subtype-specific activation-associated gene signatures of MC_T and MC_TC cells, T-type MCs were further divided into “T-type-activated MCs” and “T-type-resting MCs”, while TC-type MCs were classified into “TC-type-activated MCs” and “TC-type-resting MCs” (**Figures 3B, C**). In addition, an MC subpopulation enriched for proliferation-related genes such as MKI67, TOP2A, and PCNA was designated “Proliferative MCs” (**Supplementary Figure 2G**), which was interpreted as a transcriptionally and functionally distinct MC subset characterized by enrichment of cell cycle and proliferation associated gene signatures. Collectively, MCs were classified into five subpopulations: “T-type-activated MC”, “T-type-resting MC”, “TC-type-activated MC”, “TC-type-resting MC”, and “Proliferative MC”. Interestingly, T-type MCs were predominantly enriched in tumor tissues, whereas TC-type MCs were mainly distributed in normal tissues (**Figure 3A**). Consequently, T-type-activated MCs represented the dominant activated MC population within the ESCC TME, while TC-type-activated MCs were preferentially enriched in normal tissues. Notably, the enrichment of T-type-activated MCs in tumors largely reflected the increased abundance of the overall MC_T compartment rather than a higher activation rate within the MC_T population. These findings highlighted the marked functional heterogeneity of MC subsets in ESCC and suggested that distinct activated MC populations may play different roles within the tumor microenvironment (**Figure 3D**). Subsequently, we examined the expression patterns of MC receptors and cytokines/growth factor-related genes across these MC subpopulations (**Figure 3E**). Compared with their corresponding resting counterparts, both T-type-activated MCs and TC-type-activated MCs exhibited elevated expression of MC receptor genes, including IL1RL1 and AHR. Moreover, activated MC subsets especially TC-type-activated MCs expressed higher levels of multiple cytokines and growth factors, such as AREG, CSF1, TGFB1, CCL4, and LIF, further supporting their activated transcriptional states.

To compare the proportions of activated and resting MCs in normal and tumor tissues, we combined the activated and resting MC subsets and found that the combined activated MC subsets accounted for a higher proportion in tumor tissues, whereas the combined resting MC subsets were more abundant in normal tissues (**Supplementary Figure 2H**). To further investigate the relationship between MC subsets and T-cell infiltration within the ESCC TME, we first analyzed the correlations between TPSAB1 and CD4 as well as TPSAB1 and CD8A expression in the TCGA dataset. Significant positive correlations were observed in both analyses, suggesting a close association between MCs and T cells (**Figure 3F**). Additionally, we next evaluated the correlation between CD8^+^ T cell function and MC subsets. Interestingly, T-type MCs exhibited stronger positive associations with cytotoxicity, cytokine signaling, IFN response, and pro-apoptotic programs, whereas TC-type MCs showed relatively weaker correlations with these anti-tumor signatures. (**Figure 3G**; **Supplementary Table 7**). Together, these findings suggested that T-type MCs possessed a more immune-active transcriptional phenotype and may participate in T cell-associated immune regulation within the ESCC TME. Given that T-type-activated MCs constituted the predominant activated MC subset in tumors and were associated with immune-related and pro-apoptotic programs, these observations further implied that this MC population may potentially contribute to anti-tumor immune responses in ESCC.

### Prognosis of MCs in ESCC

**Figure 4:**
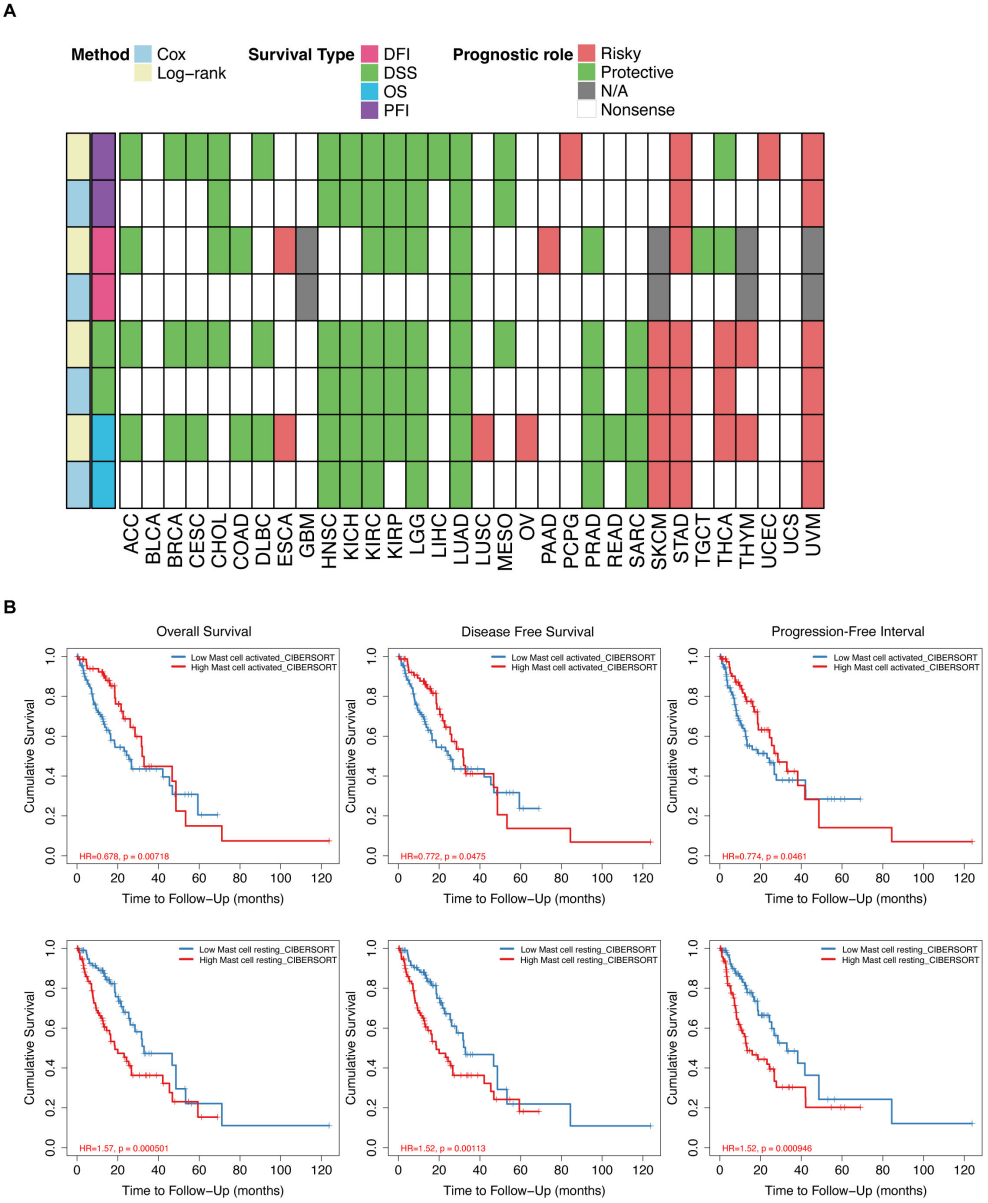
Prognosis of MCs in ESCC. **(A)** Heatmap showing the correlation between the average expression of six MC signature genes (TPSAB1, TPSB2, KIT, CPA3, HPGDS, MS4A2) and overall survival (OS), disease-specific survival (DSS), disease-free interval (DFI), and progression-free interval (PFI) on the basis of Kaplan-Meier models and univariate Cox regression. Green indicates protective factors, while red represents risk factors for patient prognosis. *P* values < 0.05. **(B)** Kaplan-Meier survival curves of activated MCs and resting MCs fraction in TCGA-ESCC dataset. All Kaplan-Meier curves above were generated using median values for grouping. The activated MCs fraction and resting MCs fraction in D were based on results obtained using CIBERSORT.

In order to explore the prognostic value of MCs in tumors, we first examined the prognostic significance of MC signature genes at the pan-cancer level using TCGA datasets. Using univariate Cox regression and Kaplan-Meier survival analyses, we evaluated the association of six MC signature genes with four key endpoints: disease-free interval (DFI), disease-specific survival (DSS), overall survival (OS), and progression-free interval (PFI). The results revealed that the six MC signature genes exerted a protective effect in 12 cancer types (including BRCA, LUAD, KIRC, and HNSC), but were associated with poorer prognosis in three cancers (SKCM, STAD, and UVM) (**Figure 4A**). Notably, no significant correlation was observed in ESCA. We reasoned that this discrepancy might reflect the limited ability of bulk RNA-seq data to resolve functionally heterogeneous MC subpopulations within the tumor microenvironment. Therefore, we further investigated the prognostic significance of distinct MC subsets in ESCC using CIBERSORT-based deconvolution analysis. Both activated and resting phenotypes were evaluated for their prognostic significance on OS, DFS, and PFI in ESCC using univariate Cox regression. Results showed that while a high percentage of resting MCs was linked to poor OS, DFS, and PFI outcomes, a high percentage of activated MCs in ESCC was linked to favorable OS, DFS, and PFI outcomes (**Figure 4B**). These findings suggested that distinct MC subsets may exert divergent biological functions within the ESCC TME and further highlighted the importance of single-cell approaches for resolving functionally heterogeneous immune cell states.

### Activation of MCs and its anti-tumor effects on ESCC

**Figure 5:**
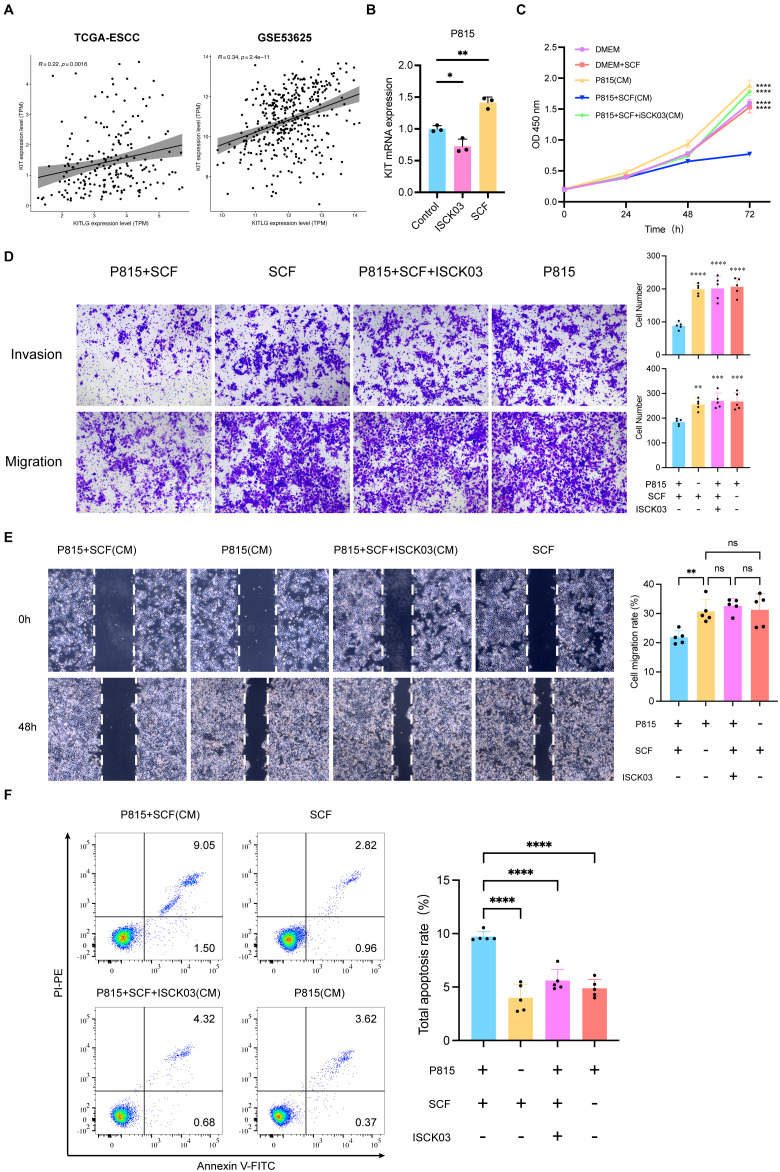
Activation of MC and its effects on ESCC. **(A)** Correlation analysis of KITLG and KIT expression in TCGA-ESCC and GSE53625 (Spearman test). **(B)** qRT-PCR analysis showed KIT mRNA expression level in P815 after co-cultured with SCF and ISCK03. **(C)** CCK-8 assays revealed the proliferative capacity of mEC25 cells cultured in supernatant (P815+SCF; P815+SCF+iSCK03; P815; DMEM; DMEM+SCF) (*n* = 5) **(D)** Transwell analysis showed the impact on mEC25 cells migration and invasion when exposed to supernatant (P815+SCF; P815+SCF+iSCK03; P815; SCF). (*n* = 5) **(E)** Wound-healing assays confirmed the migration of mEC25 cells when exposed to supernatant (P815+SCF; P815+SCF+iSCK03; P815; SCF). (*n* = 5) **(F)** The apoptosis of mEC25 cells after co-cultured by supernatant (P815+SCF; P815+SCF+iSCK03; P815; SCF) were detected by flow cytometry. (*n* = 5) All data were shown as the mean ± SD, *P* values was calculated using one-way analysis of variance. *P < 0.05; **P < 0.01; ****P < 0.0001.

Previous studies have reported that stem cell factor (SCF), also known as KIT ligand (KITLG), promotes MCs maturation and survival by inducing differentiation and migration through the c-Kit signaling pathway (32). We then examined the correlation between KIT and KITLG in the bulk RNA-seq datasets from ESCC, and revealing a significant positive association of KIT and KITLG expression (**Figure 5A**). We further used the c-Kit signaling pathway inhibitor ISCK03 to study its effect on P815 cells. P815 cells were treated with 10 μM ISCK03 and 100 ng/mL SCF for 24 hours, respectively, and Kit expression was subsequently measured by real-time RT-PCR (**Figure 5B**). The results revealed that ISCK03 reduced Kit expression, whereas SCF elevated it, confirming that SCF activates MCs through the c-Kit pathway.

We next examined the effects of activated MCs on the functional phenotypes of esophageal cancer cells *in vitro*. P815 cells were incubated with or without SCF, iSCK03. Then supernatants were collected after 24 hours and were used to treat mEC25 cells. CCK-8, transwell, and wound-healing assays were performed. Data revealed that activated MCs markedly suppressed esophageal cancer cell proliferation, migration, and invasion (**Figures 5C–E**). In addition, when mEC25 were co-cultured with SCF-treated MCs, they exhibited significantly higher apoptosis rates than other group (**Figure 5F**). Our findings indicated that SCF activates MCs, and that the resulting activated MCs may suppress tumor cell proliferation and migration while concurrently promoting tumor cell apoptosis.

### Interactions and spatial co-localization of MCs with other cells in ESCC

To further elucidate the anti-tumor mechanisms of activated MCs, we performed CellChat analysis on the integrated ESCC scRNA-seq dataset to characterize the cell-cell communication landscape within the TME. We found that both the number and intensity of interactions among cells in ESCC were greater than those in normal tissue, suggesting significant cellular activation in ESCC (**Supplementary Figure 3A**). We then comprehensively annotated cell subpopulations, incorporating both activated and resting MCs into the analysis. We quantified the number of receptors-ligand pairs between each cell type. Activated MCs displayed more interactions with other cell types compared with resting MCs (**Supplementary Figure 3B**), specially formed a great number of receptors-ligand pairs with fibroblasts, endothelial cells, and macrophages.

**Figure 6:**
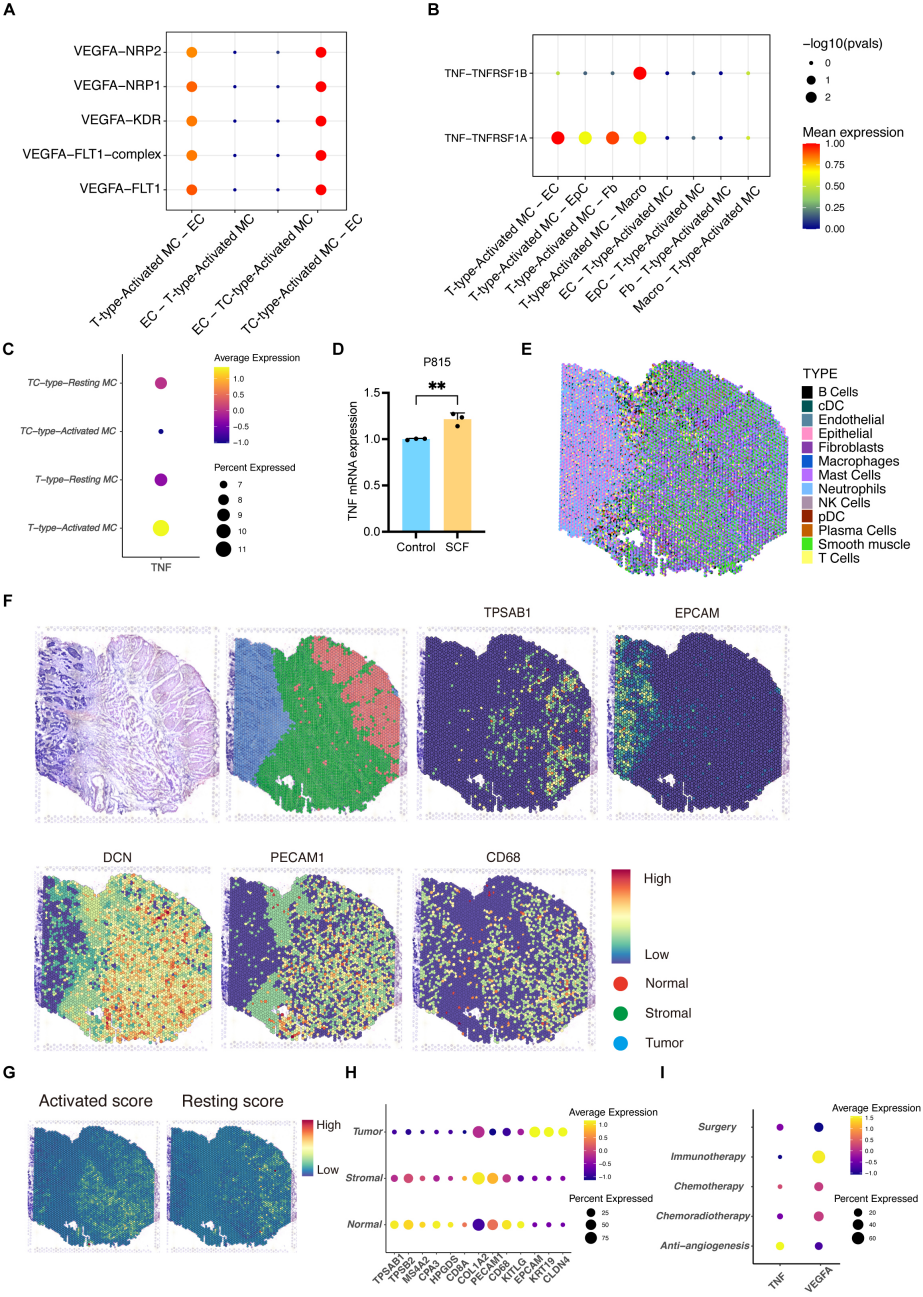
Interactions and spatial co-localization of MCs with other cells in ESCC. **(A)** Dot plot comparing VEGFA ligand-receptor signaling pathway expression between T-type-activated MCs and T-type-activated MCs. **(B)** Dot plot showing TNF ligand-receptor signaling pathway expression in T-type-activated MCs. EC: Endothelial cells; Epc: Epithelial cells; Fb: Fibroblasts; Macro: Macrophages. **(C)** Heatmap showing the expression of TNF across MC subtypes. **(D)** qRT-PCR analysis showed TNF mRNA expression level in P815 after co-cultured with SCF. *P* values were calculated by two-tailed Student’s *t*-test. **P < 0.01. **(E)** Spatial maps illustrating co-localization patterns inferred by SPOTlight. **(F)** Spatial plots of H&E staining (column 1), tissue regions (column 2), Mast cells (TPSAB1) (column 3), epithelial (EPCAM) (column 4), fibroblasts (DCN) (column 5), endothelial cells (PECAM1) (column 6), and macrophages (CD68) (column 7) in a ESCC sample. **(G)** Spatial plots of activated score and resting score of MCs. **(H)** Dot plot showing the expression of MC signatures and the regional marker genes. **(I)** Dot plot comparing the expression of TNF and VEGFA in various therapeutic interventions.

Given the marked heterogeneity observed among activated MC subsets, we next sought to determine whether distinct activated MC populations exhibited different functional signaling programs that might contribute to their divergent roles in ESCC TME. To this end, we performed CellPhoneDB analysis to investigate subtype-specific signaling pathways associated with activated MC populations. Previous studies have reported that MC-derived VEGFA was a key factor driving tumor angiogenesis. Consistent with the distinct functional programs of activated MC subsets, TC-type-activated MCs exhibited markedly stronger VEGFA-related interactions with neighboring cells than T-type-activated MCs (**Figure 6A**), suggesting that this subset may preferentially participate in angiogenic and tissue-remodeling processes. In contrast, T-type-activated MCs displayed substantially enhanced TNF signaling activity compared with the other MC subsets (**Figure 6B**). Analysis of TNF expression across the four MC subsets further demonstrated that TNF expression was highest in T-type-activated MCs, whereas both resting MC populations exhibited relatively low TNF levels (**Figure 6C**). Notably, T-type-activated MCs were preferentially enriched in tumor tissues, indicating that this activated MC subset may contribute to mainly anti-tumor immunity within the ESCC microenvironment.

Consistent with these observations, SCF stimulation of MCs enhanced their TNF expression (**Figure 6D**), suggesting that SCF-activated MCs resemble the transcriptional characteristics of T-type-activated MCs identified in the ESCC single-cell dataset. We then determined whether TNF-α released from MCs contributes to tumor cell apoptosis, anti-TNF-α neutralizing antibody was used in co-culture experiments with conditioned medium. Interestingly, we found blocking TNF-α significantly reduced apoptosis in mEC25 cells treated with conditioned medium derived from SCF-stimulated mast cells (**Supplementary Figure 3C**), suggesting that TNF-α may involve in an apoptosis-promoting effect similar to that mediated by activated MCs.

Next, to explore the spatial colocalization of MCs with other cells, we analyzed the spatial distribution of MCs in ESCC tissues using spatial transcriptomics data. We collected frozen sections of ESCC patients from Xinhua Hospital for spatial transcriptomics sequencing. Based on HE staining and gene expression information, we classified 4,157 spots into three regions: tumor, stroma, and normal. The tumor region was enriched for epithelial cell-related genes such as EPCAM, while the stroma and normal regions were enriched for fibroblast and endothelial cell-related genes (**Figure 6F**). Analysis of MCs marker gene expression revealed their predominant localization in the stroma and normal regions (**Figure 6H**). Subsequently, we annotated cell types for each spot using the deconvolution algorithm SPOTlight (**Figure 6E**). Integrating these spatial gene expression results, we found MCs primarily colocalized with fibroblasts, endothelial cells, and macrophages (**Figures 6E, F**). Also, we integrated our single-cell findings with spatial transcriptomic data, we derived gene signatures representing activated and resting mast cell subsets based on the top ten differentially expressed genes identified in single-cell analysis. To evaluate the spatial distribution of the MCs activation and resting signature, we utilized the “AddModuleScore” function in the “Seurat” package. The results showed that activated MC-associated signatures were primarily enriched in stromal regions adjacent to tumor areas, whereas resting MC-associated signatures were mainly enriched in normal tissue regions (**Figure 6G**). These findings were further validated in an independent ESCC tissue section (**Supplementary Figure 3D**), confirming the reproducibility of the spatial distribution patterns. Our results revealed that MCs are spatially co-localized with other cells in ESCC and are predominantly activated and exert their functions in regions adjacent to the tumor.

Moreover, we analyzed an ESCC dataset that included patients receiving various therapeutic interventions, such as anti-angiogenesis therapy, chemoradiotherapy, chemotherapy, and immunotherapy (**Supplementary Figure 3E**). Among all treatment groups, ESCC patients receiving anti-angiogenesis therapy showed the highest TNF expression in MCs. These findings indicate that anti-angiogenesis therapy enhances TNF expression in MCs within ESCC while concurrently suppressing VEGFA expression, thereby contributing to the inhibition of ESCC progression (**Figure 6I**).

## Discussion

Recent studies have characterized the unique characteristics of immune cells in ESCC (33–35); however, no research has yet provided an in-depth single-cell analysis of MCs in ESCC. Here, we utilized 84 samples from 73 ESCC patients, including paired tumor tissue and adjacent normal tissue to elucidate the functional complexity and role of MCs in ESCC at the single-cell level. We identified a subset of activated MCs in ESCC, these MCs correlated with high T cell cytotoxic score, which confers a protective effect on ESCC prognosis.

In this study, we first found the density of mast cells was reduced in ESCC, and then we co-cultured ESCC cell supernatants with MCs and observed increased MC apoptosis. Next, we analyzed enriched pathways for MCs in ESCC and found them highly enriched in antigen presentation and T cell activation-related pathways, a finding also reported in breast cancer (36). This suggested the presence of tumor-suppressive MCs within tumors. We then classified MCs into T-type-activated MCs, T-type-resting MCs, TC-type-activated MCs, TC-type-resting MCs, and proliferating MCs based on the expression levels of MC activation-related genes and proliferation-associated genes. We found that MC_T cells were preferentially enriched in ESCC tissues, whereas MC_TC cells predominated in normal tissues. Consequently, T-type-activated MCs represented the major activated MC population within the ESCC TME, while TC-type-activated MCs were mainly observed in normal tissues. In addition, when activation status was considered irrespective of subtype, activated MCs were more abundant in ESCC, whereas resting MCs were enriched in normal tissues. These findings highlighted the substantial functional heterogeneity of MC subsets in ESCC and suggested that distinct activated MC populations may play divergent roles in tumor progression and immune regulation. It has been reported that various signaling factors such as KITLG, IFN-γ, TGF-β, etc., are associated with the survival and functional maintenance of MCs (37). KITLG, also known as SCF, is a mast cell growth factor that activates the c-Kit pathway in MCs, promoting MC migration and enhancing MC secretory capacity, thereby activating MCs (38). KITLG and KIT expression were found to be positively correlated in the TCGA-ESCC data. We found that activated MCs could inhibit the proliferation, migration, and invasion of esophageal cancer cells while accelerating their apoptosis.

It has been reported that MC-secreted TNF-α, IL-1, and IL-6 inhibit the growth of melanoma (39). Beyond this, one of the key factors in MC-mediated tumorigenesis is angiogenesis (14, 16, 40). Based on the TNF^+^/VEGFA^+^ ratio expression levels in MCs, a recent study divided MCs into pro-tumor and anti-tumor MCs (41). In our study, we observed TNF and VEGFA expression in MC subpopulations within ESCC. We found that T-type-activated MCs highly expressed TNF. Combined with spatial transcriptomics analysis of ESCC, MCs spatially colocalized with fibroblasts, endothelial cells, macrophages, and other cells. They may exert anti-tumor effects by cooperating with other cells through TNF release. Reports indicate that IL-33-induced MCs can release large amounts of TNF-α to directly act on colon cancer cells, inhibiting their proliferation (42).

However, several limitations of the present study should be considered. Although the transcriptomic and spatial analyses were performed using human ESCC datasets, the functional validation experiments were conducted in murine cell lines. Moreover, P815 cells are derived from mastocytoma and may not fully reflect the biological properties of primary mast cells *in vivo*. Therefore, further studies using primary human MCs and *in vivo* models will be necessary to validate the antitumor functions of MCs in ESCC.

In summary, our study revealed the heterogeneity of MCs in ESCC, indicating a decrease in MC density but an increase in the proportion of activated MCs in ESCC TME. T-type-activated MCs counteract tumor growth by releasing TNF signaling, while TC-type-activated MCs highly express VEGFA may promote tumor angiogenesis. In addition, anti-angiogenic therapy can enhance TNF signaling expression. By deciphering MC heterogeneity in ESCC, our study may contribute to developing effective immunotherapies targeting MCs in ESCC.

## Data Availability

The datasets presented in this study can be found in online repositories. The names of the repository/repositories and accession number(s) can be found in the article/**Supplementary Material**. Spatial RNA-seq raw data are available from the corresponding author upon reasonable request.
