# Supplementary material for: Single cell transcriptomics analyses reveal functional heterogeneity and anti-tumor role of mast cells in esophageal squamous cell carcinoma
Source: Front Immunol. 2026 Jul 30;17:1761865. doi: 10.3389/fimmu.2026.1761865 (PMC13469595; doi:10.3389/fimmu.2026.1761865)
Supplement: Supplementary file 1 [file DataSheet1.pdf]

Single cell transcriptomics analyses reveal functional heterogeneity and anti-tumor role of mast cells in esophageal squamous cell carcinoma

Yiren Huang [1,2,3], Zheyi Chen[1], Bingqian Zhou[1], Shiyu Chen[1], Yongyu Chen[1], Qiu Pan[1], Weinan Zhu[1], Chenyu Ma[1], Shihong Chen[1], Ju Qiu[4, #], Lisong Shen [1, 2, 3, #], Yingxia Zheng [1, 2, 3, #]

1 Department of Laboratory Medicine, Xin Hua Hospital, Shanghai Jiao Tong University School of Medicine, Shanghai, China;

2 Faculty of Medical Laboratory Science, Shanghai Jiao Tong University School of Medicine, Shanghai, China.

3 Institute of Artificial Intelligence Medicine, Shanghai Academy of Experimental Medicine.

4 CAS Key Laboratory of Tissue Microenvironment and Tumor, Shanghai Institute of Nutrition and Health, University of Chinese Academy of Sciences, Chinese Academy of Sciences, Shanghai.

#Correspondence e-mail: zhengyingxia@xinhuamed.com.cn (Y.Z.); lisongshen@hotmail.com (L.S.); qiuju@sinh.ac.cn (J.Q.).

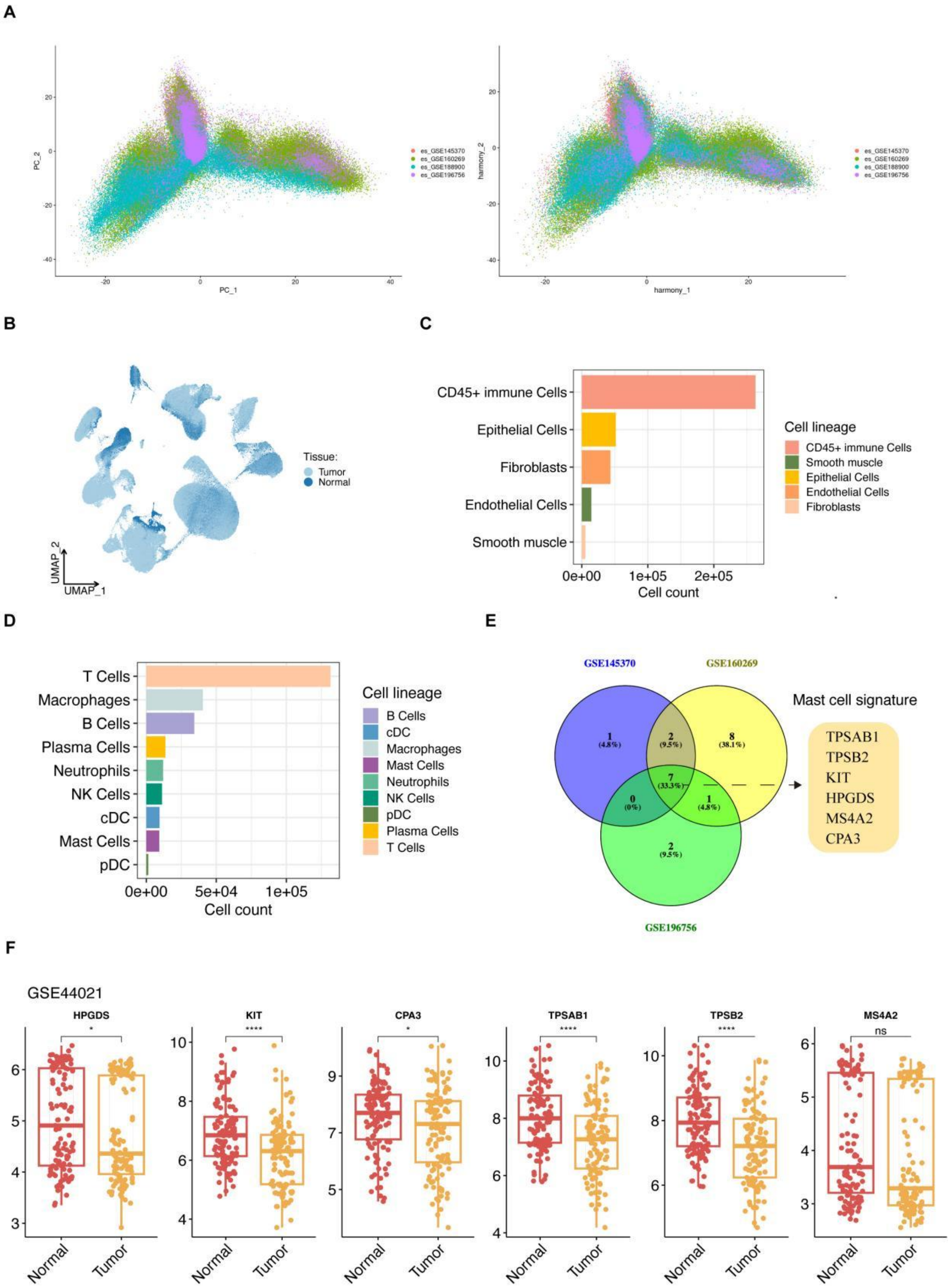

Supplementary Figure S1. (A) The batch effect correction in our study (PCA:left; Harmony:right). (B) UMAP plot of Merge_scRNA data stratified by tissue type. (C) Histogram shows the cell counts proportion of CD45$^+$ and CD45$^-$ cells. (D) Histogram shows the cell counts proportion of major CD45$^+$ cell types. (E) Venn diagram of differentially expressed genes (DEGs) of MCs in our study. (P values < 0.01,

log2FC > 1, PCT1 > 0.7, and PCT2 < 0.3). (F) Box plots showed the expression of MC marker genes in GSE44021 (Normal vs Tumor).

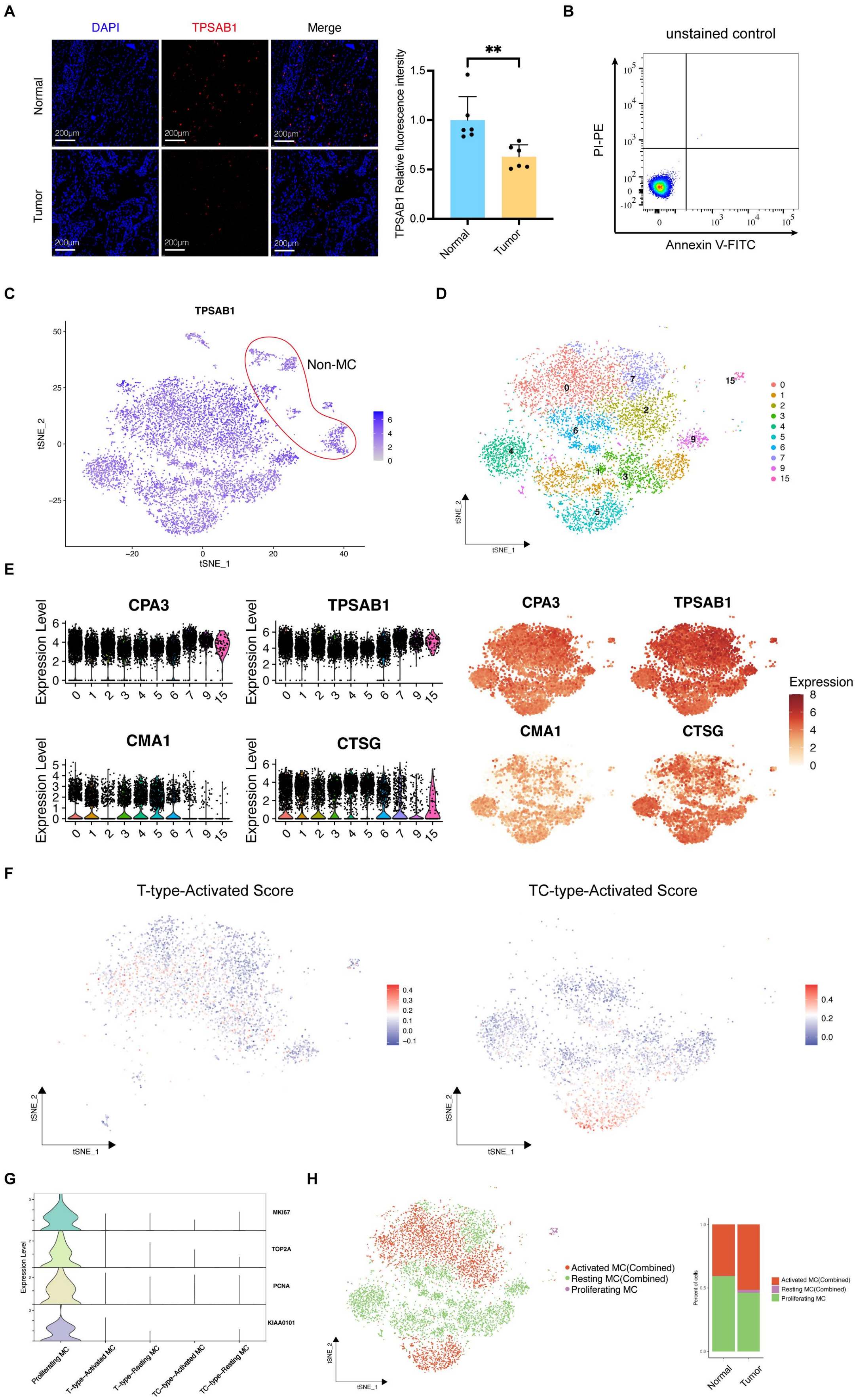
A
DAPI
TPSAB1
Merge
Normal
Tumor
200μm
TPSAB1 Relative fluorescence intensity
**
Normal
Tumor
B
unstained control
PI-PE
Annexin V-FITC
C
TPSAB1
Non-MC
tSNE_1
tSNE_2
D
tSNE_1
tSNE_2
E
CPA3
TPSAB1
CMA1
CTSG
Expression Level
Expression
F
T-type-Activated Score
TC-type-Activated Score
tSNE_1
tSNE_2
G
MKI67
TOP2A
PCNA
KIAA0101
Expression Level
Proliferating MC
T-type-Activated MC
T-type-Resting MC
TC-type-Activated MC
TC-type-Resting MC
H
Activated MC(Combined)
Resting MC(Combined)
Proliferating MC
Percent of cells
Normal
Tumor
tSNE_1
tSNE_2

Supplementary Figure S2. (A) Immunofluorescence staining of human ESCC tissue and paired NC tissue. TPSAB1 (red), DAPI (blue), Bar, 200 μm. Bar chart displayed the average fluorescence intensity of TPSAB1 between Normal and Tumor. (n=6) P values was calculated by two-tailed Student's t-test. **P<0.01. (B) Unstained control was showed to allow estimation of the true background signal. (n=5) (C) TSNE plots showed the MC subpopulation of "Non-MCs". (D) TSNE plots showed clustering of MCs with a clustering resolution of 0.8. (E) Left: Violin plot showed the expression of CPA3, TPSAB1, CMA1 and CTSG in MC clusters. Right: TSNE plots showed the expression of CPA3, TPSAB1, CMA1 and CTSG in MC clusters. (F) TSNE plots showed the activation score of T-type MC and TC-type MC. (G) Violin plot showed the expression of cell proliferation-related genes in MC clusters. (H) TSNE plots showed MCs color-coded by 3 subclusters, namely "Activated MC(Combined)", "Resting MC(Combined)", and "Proliferating MC"; Bar charts showed the proportion of MC subtypes in different tissues.

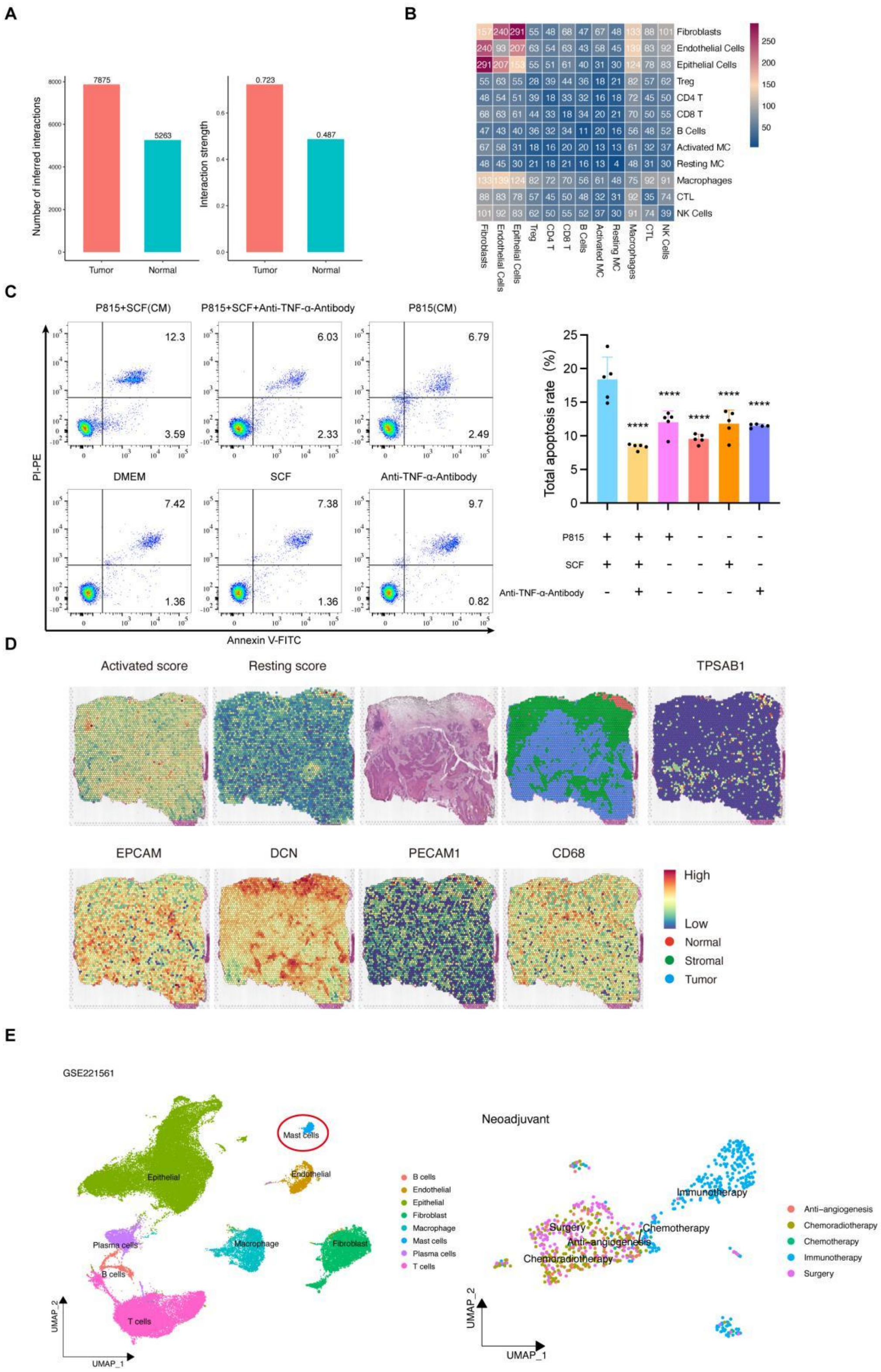

Supplementary Figure S3. (A) The histogram depicted both the number of inferred interactions and their interaction strength across major cell types, comparing normal and tumor tissues. (B) Heatmap of the number of significant ligand–receptor

interactions between major cell types and MC subtypes. (C) The apoptosis of mEC25 cells after co-cultured by supernatant (P815+SCF; P815+SCF+Anti-TNF-α-Antibody; P815; DMEM; DMEM+SCF; DMEM+Anti-TNF-α-Antibody) were detected by flow cytometry. (*n*=5). All data are shown as the mean ± SD, *P* values was calculated using one-way analysis of variance. ****P<0.0001. (D) Spatial plots of activated score (column 1), resting score (column 2), H&E staining (column 3), tissue regions (column 4), Mast cells (TPSAB1) (column 5), epithelial (EPCAM) (column 6), fibroblasts (DCN) (column 7), endothelial cells (PECAM1) (column 8), and macrophages (CD68) (column 9) in a ESCC sample. (E) UMAP plot of GSE221561 data and MCs stratified by various therapeutic interventions.
